# Measuring agency as a dimension of empowerment among young adolescents globally; findings from the Global Early Adolescent Study

**DOI:** 10.1016/j.ssmph.2019.100454

**Published:** 2019-07-19

**Authors:** Linnea A. Zimmerman, Mengmeng Li, Caroline Moreau, Siswanto Wilopo, Robert Blum

**Affiliations:** a Department of Population, Family, and Reproductive Health, Johns Hopkins Bloomberg School of Public Health, 615 N Wolfe St, Baltimore, Maryland, 21205, USA; b Faculty of Medicine, Universitas Gadjah Mada, Bulaksumur, Yogyakarta, 55281, Indonesia

## Abstract

• Agency, a domain of empowerment, is a measurable construct amongst early adolescents age 10-14.
• Three sub-scales - Voice, Freedom of Movement, and Behavioral Control and Decision-making – comprise the measurement of agency.
• Differences in the Freedom of Movement sub-scale show a growing equity gap between boys and girls across multiple countries.
• Users of the scales must consider context when adapting the items to account for differences in culture and environment.

## Background

The concept of empowerment and its links to health and well-being have gained visibility in recent years. This is reflected in the adoption of the United Nations’ Sustainable Development Goal 5 which aims to achieve gender equality and empowerment of women and girls by 2030 and global initiatives aiming to improve health and economic outcomes for women and girls worldwide through programs such as Women Deliver and Every Woman, Every Child. Cultivating empowerment and providing an enabling environment in which it can be exercised is particularly relevant for adolescents. In some contexts, entry into adolescence may be met with increasing freedom of movement and social interactions, while in others, adolescence may generate new social expectations and restrictions (Hallman et al., 2015; Mmari et al., 2018). Gender based differences may escalate with the onset of pubertal maturation, limiting aspirations and opportunities to a set of prescribed roles and exacerbating inequities by gender (Bello et al., 2017; Van eerdewijk et al., 2017). Girls may face a range of social risks and restrictions; early or forced marriage, increased home responsibilities and gender based violence. Young men may also face social pressure to adhere to traditionally masculine norms that prize aggression, risk taking, sexual drive, and the role of protector and provider (Jewkes and Morrell, 2010, 2017; Yu et al., 2017). These norms may impact health behaviors including unprotected or non-volitional sex, bullying and interpersonal violence, and substance abuse (Barker et al., 2010). Empowering adolescents, both males and females, to better negotiate these risks has the potential to reduce unsafe behaviors and carry significant positive social and health consequences in later life. As behaviors adopted during adolescence have not yet become entrenched, it is a window of opportunity – what the WHO calls “a second chance in the second decade” (Dick & Ferguson, 2015) to intervene on a number of fronts to improve health behaviors (LeCroy, 2004; Morton & Montgomery, 2013).

There has been increasing interest in programs to empower youth and thus reduce high risk behaviors and improve long-term health outcomes. Complicating the design, implementation, and evaluation of these programs, however, is a lack of clarity on how to define and measure empowerment, especially among young people. Empowerment is generally described as a process by which individuals expand their aspirations and goals and gain greater autonomy allowing them to achieve their goals (Alsop et al., 2007; Malhotra & Schuler, 2005, pp. 71–88; Narayan, 2018; Van eerdewijk et al., 2017). It is multidimensional and involves internal qualities of “agency” which represent “the capacity to make purposeful choices” (Kabeer, 1999) as well as external factors, or opportunity structures, that create the enabling environment within which individuals pursue their interests (Malhotra & Schuler, 2005, pp. 71–88).

Current research mostly focuses on women and girl's agency in late adolescence or adulthood, using proxy measures such as autonomy, voice, self-efficacy or decision making. However, research on the relevance of these constructs in early adolescence, where young people have more limited autonomy to make informed choices, is sparse. A few programs have focused on the constructs of self-efficacy and voice as strategies to empower early adolescents to make healthy decisions and improve health behaviors (Fertman & Chubb, 1992; King et al., 2002), but the results have been inconclusive (Morton & Montgomery, 2013). A different approach, including opportunity structure measures, is being tested in the Adolescent Girls Empowerment Programme (AGEP), in which empowerment is measured through social, economic and health assets. The project is currently ongoing, and though it has demonstrated some positive change in measures of self-efficacy and self-confidence, the changes appear more modest than expected and attenuated with time (Austrian et al., 2016).

Finally, the literature on measuring and defining empowerment has focused largely on women, with less attention paid to better understanding the meaning, measurement, and relationship between male and female empowerment (Kato-Wallace et al., 2016). Research has found that male engagement in reproductive health decision making, such as family planning and use of skilled delivery services, can lead to positive outcomes (Fotso, Higgins-Steele, & Mohanty, 2015; Jennings et al., 2014; Kurniati et al., 2017) and there is increasing focus on the importance of understanding and transforming gender norms (Barker et al., 2010; Dworkin, Fleming, & Colvin, 2015; Mmari et al., 2017). Yet, there is less literature on how to define and measure male's empowerment, negating the ability to assess how empowerment changes over time, across place, or in relation to female empowerment (Kato-Wallace et al., 2016). To assess how, when, and where inequities in power develop, it is critical to develop and measures that can be applied to both boys and girls.

The limited evidence of successful strategies to improve young adolescents’ autonomy in making informed choices, coupled with the global priority of enhancing empowerment, suggests the need to improve our understanding of empowerment in this age group, for both girls and boys. Building on previous frameworks, the Global Early Adolescent Study (GEAS) used formative qualitative research (Mmari et al., 2017; Saewyc, 2017) and previous measures of empowerment to develop and validate a construct of agency, relevant to the lives of early adolescents and applicable globally. This paper summarizes the construction and validation process for three sub-scales within the constrcut of agency, using data from fifteen GEAS sites. Additionally, we evaluated whether the scales varied by sex, both within and across countries, hypothesizing that within each country, scores for each sub-scale would be lower for girls than for boys.

## Methodology

### Instrument development

Multiple dimensions of empowerment have been identified in previous literature, including political, sexual and reproductive health, economic, sociological and feminist research, but not all are equally applicable to early adolescence (Cornwall, 2014; Hindin & Muntifering, 2011; Malhotra & Schuler, 2005, pp. 71–88; Narayan, 2005; Samman & Santos, 2009). A non-systematic literature review was undertaken to summarize the different conceptualizations, frameworks, and dimensions of empowerment. The review started with identifying the multiple domains that have been defined as components of women's empowerment more generally and then, through expert review, was refined to focus on the domains that were hypothesized to be relevant to young adolescents. While a major focus of empowerment research has been on women's economic empowerment (i.e. assessing the extent to which women control financial resources within a family and the decision-making power and associated outcomes that arise as a result (Buvinic, Furst-Nichols, & Courey-Pryor, 2013)), this domain was thought to be less relevant to very young adolescents and not include in the questionnaire. Similarly, women's political empowerment, focusing on issues such as the right to vote, land ownership, inheritance rights, women in political and governmental positions (Chaban, 2017), was also identified as unlikely to be relevant to 10-14 year olds.

Mobility, decision-making, and self-worth were identified by Jejeebhoy and colleagues as the three critical dimensions of empowerment for young males and females in Pune, India (Jejeebhoy et al., 2010), while agency, freedom from dominion in the household, and women's economic security were identified by Schuler and colleagues (Schuler, Hashemi, & Riley, 1997). A second dimension of agency was described by Pulerwitz and colleagues to include sexual and relationship power (Pulerwitz, Gortmaker, & DeJong, 2000). Self-efficacy, self-esteem, voice, and agency have been identified as particularly relevant concepts to adolescents and young people, though they are often measured and defined differently (Austrian et al., 2016; Fertman & Chubb, 1992; King et al., 2002) Multiple frameworks, including those published by the World Bank and the Bill and Melinda Gates Foundation were reviewed, and from these, relevant dimensions identified (Alsop et al., 2007; Malhotra & Schuler, 2005, pp. 71–88; Narayan, 2005; Van eerdewijk et al., 2017).

Following the review of the literature, which identified the dimensions and definitions above, in-depth discussions with global partners and consultations with international experts, including those from the World Bank, UNICEF, and the Young Lives Study, identified the more narrow set of constructs that were hypothesized to be directly relevant to young adolescents. From this review and consultation, three domains were identified; “Voice” (i.e. the ability to articulate choices and opinions), “Freedom of Movement” (i.e. the ability to move freely within the environment), and “Behavioral Control and Decision Making” (BCDM) (i.e. the ability to make daily decisions without adult supervision or approval).

Once identified, a search of previous surveys was undertaken to identify relevant items to include in the draft GEAS questionnaires. Multiple surveys have included items that measure constructs similar to voice including the Youth Social Self-Efficacy and the Youth Academic Self-Efficacy Scales (Muris, 2001). The scales contain items that go beyond this concept, and thus we did not use the scales in their entireties. As they were designed for youth, however, the wording and items choice influenced the subsequent development of the voice scale. Previous measures used to assess freedom of movement among adolescents came from the Caribbean Youth Health Survey (Blum et al., 2003) and the National Longitudinal Study of Adolescent to Adult Health (Resnick et al., 1997). Finally, items regarding decision making, both in daily life and in the longer term, were identified from the Health Behavior of School-Aged Children (HSBC International Coordinating Center), the Survey Assessment of Vietnamese Youth (Vietnamese Ministry of Health, 2003), the Three-City Study of Asian Adolescents and Young Adults (Zabin et al., 2012), the Well-being of Adolescents in Vulnerable Environment's Study (Sonenstein, 2014) and the American Indian – Alaska Native Youth. Relevant items within each domain were saved in a question bank and circulated to in-country partners and to expert reviewers to provide input. In-country partners provided critical insight into the cultural relevance and appropriateness of the items.

Qualitative research was also conducted in the formative phase of the GEAS to inform and improve questionnaire development [for details, see 23]. In each of 15 sites, focus groups were held with adolescents; while participants were not explicitly asked about which domains constitute empowerment, the discussions centered on how adolescents negotiate common situations with parents and peers, thus reflecting the dimensions of empowerment and providing important insights into the gendered transitions into adolescence. Several aspects of the transitions were notable, including greater autonomy in decision making for both sexes with age and restricted mobility for girls but not boys, providing confirmation that the selected domains described above were relevant for these age groups. Finally, face validity testing was undertaken with 20 respondents in each country to assess comprehension, clarity, appropriateness of answer choices and length of the survey. Feedback from the face validity was incorporated into the survey to refine the question wording and answer choices before the questonnaires were fielded as the part of Phase 1, described below.

The final items that were included in the questionnaire are listed in Table 1.

**Table 1:** Stem, item and response options by sub-domain.

Domain	Stem	Items	Response Options
Freedom of Movement	Can you tell me how often you are allowed to do the following alone (without an adult present)?	Go to after school activities (like sports clubs)	0 - Never/rarely
		Go to a party with boys and girls	1 - Sometimes
		Meet with friends after school	2 - Often
		Got to community center/movies/youth center	999 - Don't know
		Go to church/mosque/temple or religious center	996 - Refuse
		Visit a friend of the opposite sex	
Voice	How often are the following statements true for you?	My parents or guardians ask for my opinion on things	0 - Never/rarely
		My parents of guardian listen when I share my opinion	1 - Sometimes
		My friends ask my advice when they have a problem	2 - Often
		If I see something wrong in school or the neighborhood I feel I can tell someone and they will listen	999 - Don't know 996 - Refuse
		I can speak up in class when I have a comment or question	
		I can speak up when I see someone else being hurt	
		I can ask adults for help when I need it	
Behavioral control and decision making	How often are you able to make each of the following decision on your own without an adult?	What clothes to wear when you are not in school/working	0 - Never/rarely
		What to do in your free time	1 - Sometimes
		What to eat when you are not at home	2 - Often
		How much education you will get	999 - Don't know
		Who you can have as friends	996 - Refuse
		Decide when to marry on your own	
		Decide who to marry on your own	

### Sample description

This study used data from the GEAS, a large international study that focuses on early adolescents. These data come from the initial GEAS pilot, called Phase 1, which was implemented through a network of university and independent research organizations in 14 countries between November 2015 and September 2016. The GEAS Phase 1 questionnaire was administered to approximately 120 young people ages 10–14 in each country. Three countries failed to meet enrollment aims and country site was oversampled, resulting in a total sample size of 2,068 adolescents. The empowerment module was embedded in a larger survey questionnaire that included additional measures of health, gender norms and relationships (http://www.geastudy.org). After consent and assent were obtained, the questionnaires were administered either by interviewer or self-administered. The mode of interview varied among sites but was consistent within each site. All surveys were uploaded to a secure SurveyCTO^®^ server and later compiled into a single dataset for analysis.

Exploratory analysis was undertaken to determine the frequency of non-responses (do not know and refuse to answer) and construct the final analytic sample. Non-response across items ranged from 5.4% to 25.6%. Two items - when and who to marry - had non-response higher than 25% in the total sample. We thus dropped these two items from the initial scale development. Among the remaining items, 69.5% of respondents replied to all items. We dropped observations that had six or more missing responses (n = 157). For the remaining missing items, we assumed the data were missing-not-at-random and used k-Nearest Neighbor imputation (kNN) with a k-value of 38 (corresponding to the square root of complete cases) to impute missing values (Jonsson & Wohlin, 2004). The validity of imputation was assessed using exploratory factor analysis only on complete cases. Results were similar (Supplementary Table 1); to increase the sample size, we used the imputed data for analysis.

In addition, we dropped 11 respondents who did not report age or sex. In total, 7.1% of total respondents were dropped before scale construction began; 1,911 adolescents were retained for the final analysis. Table 2 shows the distribution by age, sex, and country of the original and analytic sample. The sample characteristics of the Indonesian sample are also shown below (see Table 3).

**Table 2:** Characteristics of GEAS pilot survey respondents.

	Complete sample	Analytic sample
Sexrowhead		
Female	997	983
Male	944	928
**Age**rowhead		
10	280	271
11-12	866	853
13-14	795	787
**Country**rowhead		
Ghent, Belgium	123	93
Cochabamba, Bolivia	121	112
Ougadougou, Burkina Faso	124	120
Shanghai, China	197	163
Kinshasa, DRC	123	123
Cuenca, Ecuador	115	74
Assiut, gypt	122	119
Nairobi, Kenya	560	556
Blantyre, Malawi	127	124
Ile Ife, Nigeria	122	119
Edinburgh, Scotland	36	26
Baltimore, United States	50	42
Hanoi, Vietnam	126	118
New Delhi, India	122	121
**Total**	**2068**	**1,911**

**Table 3:** Demographics for study participants from Indonesia.

	n	%
**Sex**rowhead		
Female	2160	53.28
Male	1894	46.72
**Age**rowhead		
11-12	3154	77.80
13-14	900	22.20
**Site**rowhead		
*Bandar Lampung*	1040	25.65
Female	541	52.02
Male	499	47.98
11-12	796	76.54
13-14	244	23.46
*Denpasar*	1596	39.37
Female	840	52.63
Male	756	47.37
11-12	1308	81.95
13-14	288	18.05
*Semarang*	1418	34.98
Female	779	54.94
Male	639	45.06
11-12	1050	74.05
13-14	368	25.95
**Total**	**4054**	**100**

The scales were then subsequently applied in a secondary data analysis to an external dataset from three GEAS sites in Indonesia – Bandar Lampung, Denpasar, and Semarang – to determine whether the findings from the Phase 1 study were consistent (i.e. same list of items still scale together). The data from Indonesia come from the baseline of an ongoing cohort study, which used the GEAS survey questions and methodology, and was conducted after the pilot activities described above. Indonesia did not contribute observations to the Phase 1 data used to construct the scales, but used the same tools and methodologies for data collection among boys and girls, aged 11–14, and thus constitute an external dataset on which to validate the measures. The exception to comparability is that only three ten year olds were included in the Indonesia GEAS as the sample was derived from secondary schools, which generally do not enroll students younger than 11. We validated the scales using the three sub-sites combined and by site, to assess variation across sub-sites in Indonesia.

Based on the results of the scale development, described below, we chose to assess each sub-scale separately. We dropped observations that had 40% or more missingness across items within each subscale and then used kNN imputation to impute remaining missing values. Sensitivity analyses were conducted to compare results from the imputed and complete case data. As results were comparable and the retained sample sizes for complete cases within each sub-scale were large, we chose to retain only complete cases. From a total of 4,684 observations, 3,604 observations were retained for the Freedom of Movement scale, 3,404 for Voice, and 3,296 for BCDM.

### Ethical clearance

The World Health Organization Ethical Review Board, the Johns Hopkins Bloomberg School of Public Health IRB, and each site's human subject ethics review committee approved all research protocols.

### Analysis

Following exploratory analysis, we conducted exploratory factor analysis, retaining all items to determine if the three hypothesized factors were present. We kept all factors with eigenvalues greater than 1 and these extracted factors were rotated using promax rotation (factor correlation > 0.30) to retrieve the factor loadings. Three factors were retained in the final model, corresponding to each of the above mentioned constructs – Freedom of Movement, Voice, and Behavioral Control and Decision Making (BCDM). Items for which the factor loading was lower than 0.40 in all three factors were eliminated. Subscale analysis for each of the three constructs was conducted by country to further refine the scales. Sub-scale internal reliability was measured using polychoric ordinal alpha reliability coefficient. Sample adequacy was consisitently evaluated using Kaiser-Meyer-Olkin (KMO) Test, and we adhered to the published standards to assess adequacy (Kaiser, 1974). Sensitivity analyses conducted using only the sample with complete responses to all items showed no significant differences between the imputed and complete case datasets.

Mean scores were calculated based on each set of scaled items, which ranged from 1 to 4. Scale-based mean scores were calculated, across scaled items, as the average of summed scores of items, which individually was weighted by its corresponding factor loading. In Indonesia, though the observations for ten year olds were included in the factor analysis, we did not compute mean scores for this age group given the lack of sample adequacy. T-tests and linear regression with categorical independent variables were used to determine if differences in mean scores by group were statistically significant.

All analyses were conducted on Stata SE version 15.1, StataCorp LLC, TX and R Version 3.6.0 (R Project).

## Results

Table 4 shows the three factors and factor loading of the items using the pilot data. All of the items loaded onto the expected factor and none loaded onto two factors with a factor loading greater than 0.28 for the second factor, indicating that they measure distinct concepts. The factor representing Voice accounted for the largest percentage of variation in the scale (43.0%) followed by Freedom of Movement and BCDM (34.8% and 29.8%, respectively). The ordinal alpha for each sub-scale was between 0.79 (95% CI: 0.78-0.81) and 0.86 (95% CI: 0.85-0.87). One item, whether the respondent could travel to a religious center alone, was dropped from the Freedom of Movement sub-scale as it did not load at 0.40 on any scale and only 4% of the variance in the item was shared with other variables (values not shown). Once this item was removed, the overall ordinal alpha for the Freedom of Movement score increased from 0.72 to 0.83.

**Table 4:** Factor Loadings for Empowerment Scale Items: combined results from kNN-imputed data (n = 1911).

Item	Voice	Freedom of Movement	Behavioral Control and Decision Making	Uniqueness
Go to after school activities (like sports clubs)	0.03	0.50	0.21	0.60
Go to a party with boys and girls	-0.10	0.83	-0.01	0.38
Meet with friends after school	0.09	0.54	0.10	0.60
Got to community center/movies/youth center	0.03	0.80	-0.03	0.35
Visit a friend of the opposite sex	0.05	0.73	-0.04	0.47
My parents or guardians ask for my opinion on things	0.58	0.14	0.01	0.57
My parents of guardian listen when I share my opinion	0.68	0.01	0.02	0.52
My friends ask my advice when they have a problem	0.63	0.06	0.04	0.54
If I see something wrong in school or the neighborhood I feel I can tell someone and they will listen	0.71	0.04	-0.06	0.52
I can speak up in class when I have a comment or question	0.68	-0.12	0.08	0.52
I can speak up when I see someone else being hurt	0.74	0.02	-0.06	0.48
I can ask adults for help when I need it	0.65	-0.09	0.04	0.59
What clothes to wear when you are not in school/working	-0.02	-0.03	0.73	0.50
What to do in your free time	0.00	0.10	0.71	0.43
What to eat when you are not at home	-0.05	0.11	0.67	0.51
How much education you will get	0.14	-0.09	0.48	0.72
Who you can have as friends	0.05	-0.14	0.66	0.58
Ordinal Alpha (95% CI)	0.86 (0.85, 0.87)	0.83 (0.82, 0.84)	0.79 (0.78, 0.81)	
Eigenvalue	3.18	2.58	2.20	
Total % of Variance Explained	42.97	34.80	29.81	
Sample Adequacy (KMO value)	0.89			

Country specific results are shown in Table 5 for countries that had a minimum of 100 observations.

**Table 5:** Factor Loadings fo Sub-Scale Items: country specific results from kNN-imputed data.

Voice		Bolivia		Burkina Faso		China		DRC		Egypt		Kenya	Malawi		Nigeria	Vietnam		India
**Voice**		n = 112		n = 120		n = 163		n = 123		n = 119		n = 556	n = 124		n = 119	n = 118		n = 121
My parents or guardians ask for my opinion on things		0.62		0.52		0.82		0.59		0.79		0.43	0.61		0.81	0.58		0.49
My parents of guardian listen when I share my opinion		0.65		0.48		0.90		0.80		0.48		0.67	0.79		0.71	0.57		0.59
My friends ask my advice when they have a problem		0.62		0.66		0.87		0.57		0.69		0.59	0.74		0.44	0.46		0.81
If I see something wrong in school or the neighborhood I feel I can tell someone and they will listen		0.70		0.73		0.73		0.60		0.55		0.65	0.65		0.78	0.55		0.69
I can speak up in class when I have a comment or question		0.68		0.52		0.74		0.58		0.54		0.69	0.75		0.63	0.69		0.89
I can speak up when I see someone else being hurt		0.68		0.58		0.83		0.59		0.58		0.75	0.72		0.56	0.65		0.64
I can ask adults for help when I need it		0.55		0.74		0.64		0.53		0.41		0.54	0.81		0.62	0.53		0.84
Ordinal Alpha (95% CI)		0.83 (0.78, 0.88)		0.80 (0.74, 0.85)		0.92 (0.90, 0.94)		0.80 (0.74, 0.85)		0.77 (0.71, 0.84)		0.81 (0.79, 0.84)	0.89 (0.86, 0.92)		0.83 (0.79, 0.88)	0.77 (0.71, 0.84)		0.86 (0.82, 0.90)
Sample Adequacy (KMO value)		0.80		0.80		0.90		0.71		0.72		0.82	0.86		0.67	0.64		0.75
**Freedom of Movement**rowhead																		
Go to after school activities (like sports clubs)	0.46		0.62		0.68		0.50		0.49		0.48		0.71	0.63		0.63	0.82	
Go to a party with boys and girls	0.63		0.72		0.85		0.66		0.43		0.71		0.63	0.88		0.70	0.77	
Meet with friends after school	0.63		0.76		0.76		0.52		0.43		0.49		0.67	0.64		0.60	0.76	
Go to community center/movies/youth center	0.81		0.99		0.74		0.85		0.94		0.74		0.51	0.89		0.95	0.53	
Visit a friend of the opposite sex	0.78		0.83		0.64		0.69		0.46		0.64		0.37	0.81		0.81	0.75	
Ordinal Alpha (95% CI)	0.77 (0.70, 0.84)		0.89 (0.85, 0.92)		0.85 (0.81, 0.89)		0.78 (0.71, 0.84)		0.67 (0.57, 0.76)		0.76 (0.72, 0.79)		0.71 (0.63, 0.79)	0.88 (0.84, 0.91)		0.83 (0.78, 0.88)	0.82 (0.77, 0.87)	
Sample Adequacy (KMO value)	0.58		0.82		0.83		0.76		0.49		0.73		0.65	0.85		0.81	0.67	
**Behavioral Control and Decision Making (BCDM)**rowhead																		
What clothes to wear when you are not in school/working	0.79		0.88		0.76		0.48		0.43		0.72		0.71	0.50		0.69	0.78	
What to do in your free time	0.74		0.62		0.93		0.54		0.64		0.70		0.55	0.75		0.89	0.80	
What to eat when you are not at home	0.57		0.71		0.86		0.48		0.80		0.63		0.52	0.53		0.65	0.80	
How much education you will get	0.38		0.45		0.53		0.41		0.59		0.51		0.47	0.46		0.16	0.59	
Who you can have as friends	0.58		0.67		0.73		0.65		0.67		0.56		0.48		0.67	0.45	0.48	
Ordinal Alpha (95% CI)	0.75 (0.67, 0.82)		0.80 (0.74, 0.86)		0.87 (0.84, 0.90)		0.64 (0.53, 0.74)		0.76 (0.70, 0.83)		0.76 (0.73, 0.79)		0.69 (0.60, 0.78)		0.72 (0.64, 0.80)	0.68 (0.60, 0.77)	0.82 (0.77, 0.87)	
Sample Adequacy (KMO value)	0.64		0.74		0.84		0.67		0.71		0.78		0.58		0.70	0.71	0.62	

The Voice sub-scale had the highest alpha of the three scales in seven countries. There was variation across countries in the factor loading of each item in the sub-scale, but all items loaded at least at 0.40 in every country. The Freedom of Movement sub-scale had the highest alpha among the three scales in Burkina Faso, Nigeria, and Vietnam. Only in one country, Egypt, did the Freedom of Movement scale perform significantly better with the inclusion of the item related to travel to a religious center (not shown). The BCDM Scale had the lowest internal reliability of the three scales, and was below 0.70 in the Democratic Republic of the Congo, Malawi, and Vietnam. In general, the item related to education attainment had the lowest factor loading and relevance to the overall dimensionality, though it retained relatively high loadings in Egypt and India.

Finally, we calculated the mean value for each sub-scale and the overall scale by age group, sex, and country (Table 6). For both the overall scale and sub-scales, girls had statistically significantly lower mean scores than boys. The widest difference was in the Freedom of Movement scale, where the average score of boys was 2.09 and for girls 1.77 (p < .01). For all scales, there was a positive trend towards increasing mean scores with age. For voice, behavioral control, and the overall scale, the mean scores for 11–12 and 13-14 year olds were higher than for 10 year olds (p < .01). While, there was no difference in mean scores of Freedom of Movement comparing 10 with 11-12 year olds (1.85 and 1.89, respectively), there was a statistically significant difference between 10 and 13-14 years of age (1.85 and 1.99, respectively).

**Table 6:** Mean scores for sub-scale and total scale by age, gender and country/gender.

		Voice	Freedom of Movement	Behavioral Control and Decision Making
Rangerowhead				
Mean		2.85	1.92	2.75
Min		1.25	1.15	1.17
Max		3.76	3.45	3.50
Sexrowhead				
Boy		2.92	2.09	2.78
Girl		2.78***	1.77***	2.72**
Agerowhead				
10 (ref)		2.67	1.85	2.64
11-12		2.88***	1.89	2.75***
13-14		2.89***	1.99***	2.79***
Country:rowhead				
Bolivia	Boy	2.79	1.98	2.87
	Girl	2.74	1.52***	2.98
Burkina Faso	Boy	2.96	2.22	2.76
	Girl	2.87	1.28***	2.81
China	Boy	2.47	1.92	**2.53**
	Girl	2.51	1.96	2.73*
DRC	Boy	2.26	1.84	2.78
	Girl	2.42	1.67*	2.55**
Egypt	Boy	3.09	2.70	2.94
	Girl	2.53***	1.97***	2.44***
Kenya	Boy	3.00	1.82	2.60
	Girl	2.81***	1.64***	2.52*
Malawi	Boy	3.28	2.65	3.29
	Girl	3.18	2.45*	3.22
Nigeria	Boy	3.24	1.73	2.96
	Girl	2.91***	1.31***	2.79*
Vietnam	Boy	3.03	1.81	2.68
	Girl	3.18	1.59**	2.62
India	Boy	3.26	2.70	3.30
	Girl	2.70***	2.10***	3.07***

*p < .10, **p < .05, ***p < .01.

The Freedom of Movement sub-scale demonstrated the most divergence between boys and girls; every site, other than China, had moderately to strongly statistically significantly lower mean scores for girls than for boys. The largest differences were seen in Burkina Faso and Egypt. In Egypt, Kenya, Nigeria, and India, girls had statistically significantly lower mean scores than boys for Voice; the largest differences were in Egypt and India.

Sex differences were less pronounced for decision making. The largest differences were again found in Egypt and India, however, Democratic Republic of the Congo, Kenya and Nigeria showed large differences between boys and girls. China was the only country where the mean BCDM score for girls was higher than boys, though the difference is modest (p < .10).

Factor loadings for the three sub-scales in Indonesia are shown in Table 7. Between the original fielding of the GEAS pilot questionnaire and data collection in Indonesia, one of the question items in the BCDM scale – “How much education you will get” - was changed to “How much do you think you will influence the decision when to leave to school”. The verbage change affected the factor loadings significantly and we removed the item from the Indonesia specific analysis.

**Table 7:** Indonesia specific factor loadings and uniqueness for each sub-scale, overall and by site.

Voice	Indonesia (N = 3,404)		Bandar Lampung (N = 788)		Denpasar (N = 1407)		Semarang (N = 1209)	
	Factor Loading	Uniqueness	Factor Loading	Uniqueness	Factor Loading	Uniqueness	Factor Loading	Uniqueness
My parents or guardians ask for my opinions on things	**0.64**	0.59	**0.69**	0.52	**0.65**	0.57	**0.58**	0.67
My parents or guardians listen when I share my opinions	**0.69**	0.52	**0.73**	0.47	**0.74**	0.45	**0.60**	0.64
My friends ask my advice when they have a problem	**0.71**	0.49	**0.77**	0.41	**0.71**	0.50	**0.67**	0.56
If I see something wrong in school or the neighborhood I feel I can tell someone and they will listen	**0.76**	0.42	**0.82**	0.33	**0.76**	0.43	**0.72**	0.48
I can speak up in class when I have a comment or question	**0.70**	0.51	**0.74**	0.45	**0.73**	0.47	**0.63**	0.60
I can speak up when I see someone else being hurt	**0.74**	0.46	**0.79**	0.38	**0.72**	0.48	**0.70**	0.50
I can ask adults for help when I need it	**0.73**	0.47	**0.78**	0.39	**0.71**	0.50	**0.70**	0.50
***Ordinal Alpha (95% CI):***	*0.88 (0.87, 0.88)*		*0.91 (0.89, 0.92)*		*0.88 (0.87, 0.89)*		*0.84 (0.83, 0.86)*	
***Eigenvalue***	*4.03*		*4.47*		*4.09*		*3.61*	
***Total % of Variance Explained***	*57.63%*		*63.79%*		*58.45%*		*51.52%*	
***Sample Adequacy (KMO value)***	*0.87*		*0.88*		*0.88*		*0.85*	
Freedom of Movement	Indonesia (N = 3,604)		Bandar Lampung (N = 905)		Denpasar (N = 1443)		Semarang (N = 1256)	
	Factor Loading	Uniqueness	Factor Loading	Uniqueness	Factor Loading	Uniqueness	Factor Loading	Uniqueness
Go to after-school activities (like sports clubs)	**0.49**	0.76	**0.54**	0.71	**0.48**	0.77	**0.47**	0.78
Go to a party with boys and girls	**0.77**	0.41	**0.84**	0.29	**0.73**	0.46	**0.75**	0.44
Meet with friends after school	**0.53**	0.72	**0.50**	0.75	**0.61**	0.63	**0.48**	0.77
Go to community center/movies/youth center	**0.66**	0.56	**0.72**	0.48	**0.68**	0.54	**0.60**	0.64
Visit a friend of the opposite sex	**0.67**	0.55	**0.73**	0.46	**0.65**	0.58	**0.64**	0.59
***Ordinal Alpha (95% CI):***	*0.76 (0.75, 0.77)*		*0.80 (0.78, 0.82)*		*0.76 (0.74, 0.78)*		*0.72 (0.70, 0.75)*	
***Eigenvalue***	*2.58*		*2.80*		*2.59*		*2.39*	
***Total % of Variance Explained***	*51.50%*		*56.05%*		*51.75%*		*47.73%*	
***Sample Adequacy (KMO value)***	*0.77*		*0.78*		*0.76*		*0.75*	
Behavioral Control and Decision Making	Indonesia (N = 3,296)		Bandar Lampung (N = 749)		Denpasar (N = 1358)		Semarang (N = 1189)	
	Factor Loading	Uniqueness	Factor Loading	Uniqueness	Factor Loading	Uniqueness	Factor Loading	Uniqueness
What clothes to wear when you are not in school/working	**0.42**	0.82	**0.50**	0.75	**0.42**	0.82	*-dropped-*	
What to do in your free time	**0.82**	0.33	**0.82**	0.32	**0.83**	0.31	**0.77**	0.40
What to eat when you are not at home	**0.76**	0.43	**0.78**	0.39	**0.76**	0.43	**0.75**	0.44
Who you can have as friends	**0.70**	0.51	**0.80**	0.36	**0.70**	0.52	**0.65**	0.58
***Ordinal Alpha (95% CI):***	*0.76 (0.75, 0.78)*		*0.81 (0.79, 0.84)*		*0.77 (0.74, 0.79)*		*0.72 (0.69, 0.74)*	
***Eigenvalue***	*2.37*		*2.59*		*2.38*		*2.04*	
***Total % of Variance Explained***	*59.33%*		*64.86%*		*59.53%*		*68.00%*	
***Sample Adequacy (KMO value)***	0.75		0.78		0.75		0.69	

As with the original scale development, the Voice sub-scale had the highest ordinal alpha among the three scales, ranging from 0.84 (95% CI: 0.83-0.86) in the Semarang sample to 0.91 (95% CI: 0.89-0.92) in the Bandar Lampung sample. There was a consistent pattern of factor loadings across the three sites, with the lowest factor loading associated with the item “my parents ask for my opinion on things” and the highest associated with “If I see something wrong in school or my neighborhood, I can tell someone and they will listen”, although the levels of the factor loadings varied by sub-site. The Freedom of Movement scale and BCDM scale had similar alpha values in all sites, ranging from a high of 0.80 (95% CI: 0.78-0.82) and 0.81(95% CI: 0.79-0.84), respectively, in Bandar Lampung to a low of 0.72 (95% CI: 0.70-0.75) and 0.72 (95% CI: 0.69-0.74) in Semarang. Again, the items within each sub-scale followed a similar pattern in the factor loadings across sites, although the values differed. In the Freedom of Movement scale, meeting friends or going to after school activities loaded lowest, while items related to visiting a friend of the opposite sex or going to parties with boys and girls loaded highest. One item in the BCDM scale – related to how often the respondent could choose what clothes to wear - did not achieve a factor loading higher than 0.40 in Semarang and was dropped in that site, while it had the lowest factor loading in the other two sites.

Differences in age were less pronounced in the three sites in Indonesia than in the pilot study sites (Table 8), but were present by sex in all three sites and in the total sample. Contrary to the pilot data, girls had statistically higher mean scores than boys for the voice and decision making sub-scales across all three sites, with the exception of BCDM scale in Denpasar. Consistent with the pilot data, girls in all three sites had statistically significantly lower mean scores for the Freedom of Movement sub-scale.

**Table 8:** Weighted mean scores for sub-scales by age and sex in Indonesia, total sample and by site.

	Voice			
	Overall	Bandar Lampung	Denpasar	Semarang
Rangerowhead				
Mean	2.11	2.23	2.15	1.95
Min	0.71	0.76	0.72	0.66
Max	2.84	3.04	2.87	2.63
Agerowhead				
11-12 (ref)	2.11	2.24	2.16	1.94
13-14	2.09	2.19	2.13	1.95
Sexrowhead				
Boy	2.05	2.16	2.08	1.89
Girld	2.15^∗∗∗,^a	2.28^∗∗∗,^^a^	2.21^∗∗∗,^^a^	1.99^∗∗∗,^^a^
	Freedom of Movement			
	Overall	Bandar Lampung	Denpasar	Semarang
Rangerowhead				
Mean	1.37	1.43	1.40	1.28
Min	0.63	0.67	0.63	0.59
Max	2.50	2.67	2.51	2.35
Agerowhead				
11-12 (ref)	1.36	1.41	1.40	1.28
13-14	1.39	1.49^∗∗^	1.39	1.29
Sexrowhead				
Boy	1.43	1.52	1.46	1.32
Girl	1.32^∗∗∗,^^a^	1.34^∗∗∗,^^a^	1.35^∗∗∗,^^a^	1.25^∗∗∗,^^a^
	Behavioral Control and Decision-making			
	Overall	Bandar Lampung	Denpasar	Semarang
Rangerowhead				
Mean	1.98	2.08	1.99	2.09
Min	0.67	0.73	0.68	0.70
Max	2.70	2.91	2.71	2.79
Agerowhead				
11-12 (ref)	1.98	2.08	2.00	2.09
13-14	1.96	2.06	1.96	2.07
Sexrowhead				
Boy	1.95	2.04	1.97	2.04
Girl	2.00^∗∗∗,^^a^	2.12^∗∗,^^a^	2.01	2.13^∗∗∗,^^a^

*p < .10; **p < .05; ***p < .01.

a = Student t-test.

## Discussion

The central intent of this work was to assess whether three dimensions of agency in this age group-- voice, freedom of movement, and behavioral control and decision making-- were measurable and formed a valid concept among adolescents age 10–14. Across sites, each sub-scale had high internal consistency and contributed substantially to explaining the total variance in measurement for both boys and girls. These results were largely consistent in an external dataset, composed of three sites in Indonesia. Of the three sub-scales, voice appeared to be the most universal construct, with an ordinal alpha above .70 in all countries and the highest alpha across all Indonesia sites. Conceptually, the fact that voice is the strongest of the three subscales is consistent with other models of empowerment where voice is often central to the definition of empowerment and in some frameworks has been included as a separate domain (Scales, Benson, & Roehlkepartain, 2011; Van eerdewijk et al., 2017). The ordinal alpha for the Freedom of Movement and Behavioral Control and Decision Making varied more by site. It may be that the items underpinning these concepts are less universal and require more contextualized adaptation. Context is critical to measuring empowerment, as the manifestations of power may differ significantly across contexts (Richardson, 2018; Samman & Santos, 2009). For example, the freedom to visit a doctor alone has been shown to be a sign of empowerment for women in some contexts (where traveling alone is uncommon) but less meaningful in others (Malhotra & Schuler, 2005, pp. 71–88). While the measure we have developed works well across geographic locals, the country specific analyses and the secondary analysis in Indonesia demonstrate the important role that context plays in defining the behaviors and attitudes that meaningfully compose agency.

Country specific analyses showed considerable variation in the factor loadings of items. The example of the ability to choose clothing in Indonesia underscores the critical role of understanding context when including and interpreting scale items. While the inclusion of this item in the total sample contributed substantially and had high factor loadings in several sites, its contribution to the scale was much lower in Indonesia, and particularly in one site, where students are expected to wear student uniforms. In Vietnam, as in other countries with universal secondary education, high education expectations, and nearly universal high educational attainment for both sexes, this question may be less relevant than in others where expectations may be lower. Thus, context and cultural norms will affect the relative importance of each item and should be considered when adopting and adapting items.

Though we found that these constructs worked well in the majority of countries, the sub-scale of Behavioral Control and Decision Making did not seem to scale well in DRC or Malawi. Although the score was low in other countries, generally removing one item that loaded poorly within each country improved the fit. In DRC and Malawi, however, none of the items stand out as particularly high or low loading items. A more detailed analysis of the Malawi data showed less variation in both the BCDM and the Freedom of Movement items and generally higher scores across the items relative to other countries. This was not the case in Democratic Republic of the Congo where no one answer choice accounted for more than 50% of any item. Similarly, this scale was the only one where the removal of a specific item substantially imporved the fit in the data in Indonesia. It is likely that the daily decisions that are allowed to adolescents vary across sites and thus a better understanding the contextual factors that influence the dimensions of empowerment is necessary before adapting each scale.

The differences in scores by age and sex are consistent with the conceptualization of the three dimensions of agency. For a variety of reasons, younger adolescents may be less able to exercise agency relative to older adolescents; cognitively, the youngest adolescents may be less able to make decisions among a range of options and/or may be more likely to be restricted in their movements than older peers. Overall, we found that the three dimensions of agency applied to both boys and girls. The introduction of a measure of agency that can be applied to both sexes contributes substantially to the field, as the measurement of male empowerment and agency remains nascent and thus limits our ability to assess inequity between sexes and over time (Kato-Wallace et al., 2016). The variation in scores across all domains by sex is in keeping with the literature that by early adolescence differences in gender expectations intensify (Hallman et al., 2015; Mmari et al., 2018). This difference is particularly pronounced for Freedom of Movement, where in every country other than China, girls had statistically significantly lower scores than boys. The differences in scores across countries for Freedom of Movement likely also reflect context, wherein some sites may be more safe to navigate alone, regardless of sex. Significant differences between sexes within countries cannot be exclusively explained by the safety of the environment, however. Rather, this points towards differences in regulating and constraining movement among young females more so than males, confirming previous qualitaive findings (Bello et al., 2017; Mmari et al., 2018). In unsafe areas, these differences may be amplified. Future research should explore how agency changes by age within sexes and, as adolescents age and new dimensions of agency and empowerment become relevant, new measures, that include males, may be needed to assess differences between sexes.

There are limitations to this study that should be considered. Prior to the initial development of the questionnaire and the empowerment module, we did not conduct a systematic literature review, instead relying on a broad search of the literature and expert review to determine items best suited for inclusion. The broad themes that were idenified through this process, however, were echoed in the qualitive work that grounded the questionnaire and provide additional support to our hypothesis that these constructs can be measured amongst very young adolescents. Though the overall sample size was large, with the exception of Nairobi, each country specific site had limited sample sizes. In some cases, this affected sample adequacy, particularly for the Freedom of Movement scale in Egypt. Small sample sizes was additionally problematic in two of the high income settings - Scotland and the United States – which precluded our ability to investigate the site specific propreties of the subscales in settings where agency and empowerment may manifest differently. Despite the limitations in sample size and exclusion of some high income sites, the scores across the three sites in Indonesia indicate that these scales perform well in an external dataset. Of note, there were only three ten year olds included in the Indonesian dataset, which limited our ability to draw conclusions about how this group differs from older adolescents. Finally, the degree of variation in answer choices was limited to only three options. Scale development is generally improved by increasing the number of response options, however, we felt that in order to limit respondent fatigue and improve comprehension amongst the youngest adolescents, a limited set of options was preferable.

Despite these limitations, the study has a number of strengths. First, the questionnaire was developed through engagement with stakeholders from diverse countries as well as with international empowerment researchers to ensure that the items were relevant in the majority of contexts. The items comprising the empowerment measure were initially developed by those with expertise in early adolescent development to ensure that the items were comprehensible to adolescents of this age group. The measure was also piloted twice in culturally diverse sites and applied to an external dataset. To our knowledge, the data reported here represent the largest and most diverse sample of young adolescents to be included in the development of an empowerment measure.

Because of the global focus on empowerment, it is critical to develop and refine measurement for adolescents, both boys and girls. Future work should explore the interplay between opportunity structures and the identified dimensions of agency. The GEAS tools include a range of measures exploring the ecological influences shaping young people's lives that enable or constrain young people's choices. However, the GEAS provides relatively little information on the larger political and legal structures that influence the ability of adolescents to make and achieve choices and goals. Some structures will not be directly relevant to young adolescents or amenable to program change, but it is worthwhile to identify those that are and further, those that are measurable by surveys, to understand how these and other contextual and structural factors impact choice and decision making. This should be supplemented with additional research on the role of parents in facilitating or limiting adolescents' agency, as most adolescents must navigate the expression and achievement of their choices through their relationships with their parents and caregivers. Finally, while these concepts demonstrate a general measurement of agency, empowerment and agency can also be expressed in highly specific domains which may vary between boys and girls (Samman & Santos, 2009) and may be expressed through additional dimensions that we have not included here. Empowerment within the realm of sexual and reproductive health, for example, may require more specific measurement than the general domains of agency measured in GEAS. While research has been conducted in this sphere (Corroon et al., 2014; Hindin & Muntifering, 2011; Upadhyay et al., 2014), less work has been done to adapt measures for young adolescents who will shortly enter their reproductive years. Ensuring that these measures are developed and can be applied to both boys and girls will improve our ability to identify when and how inequities arise and create interventions accordingly.

## Conclusions

Our analysis has demonstrated that the concept of agency, as defined by voice, freedom of movement and decision making, is measurable amongst adolescents 10–14 globally. The patterns across age and sex are in keeping with other literature that demonstrates a growing equity gap and reinforced gender norms in later adolescence. Despite the universality of the general concept, agency, and empowerment more broadly, is complex to define and measure. Context is critical; access to resources, family dynamics, community, and cultural norms all influence how agency can be measured (influencing item selection and wording) and how it can be expressed on a larger scale. It will be critically important in future research to better understand the contextual factors and the opportunity structures that are most influential and relevant to young adolescents and through which agency operates.

## Ethical statement

Ethical approval was granted by the Johns Hopkins Bloomberg School of Public Health Institutional Review Board, the World Health Organization Ethical Review Board and the relevant partner's national ethics committees.

## Declarations of interest

None.
